# Visceral Fat Accumulation Is Associated with Asthma in Patients with Type 2 Diabetes

**DOI:** 10.1155/2019/3129286

**Published:** 2019-05-02

**Authors:** Daisuke Murakami, Futoshi Anan, Takayuki Masaki, Yoshikazu Umeno, Takehiko Shigenaga, Nobuoki Eshima, Takashi Nakagawa

**Affiliations:** ^1^Department of Otorhinolaryngology, Graduate School of Medical Sciences, Kyushu University, Fukuoka, Japan; ^2^Department of Cardiology, Oita Red Cross Hospital, Oita, Japan; ^3^Department of Endocrinology, Metabolism, Rheumatology and Nephrology, Faculty of Medicine, Oita University, Oita, Japan; ^4^Department of Endocrinology, Oita Red Cross Hospital, Oita, Japan; ^5^Department of Pulmonary, Oita Red Cross Hospital, Oita, Japan; ^6^Center for Educational Outreach and Admissions, Kyoto University, Kyoto, Japan

## Abstract

**Objective:**

The number of patients with type 2 diabetes has increased in Japan, and type 2 diabetes has attracted attention as a risk factor for asthma. However, the risk factors for the development of asthma in patients with type 2 diabetes have not been determined. This study was performed to clarify whether visceral fat accumulation (VFA) and insulin resistance are risk factors for the development of asthma in patients with type 2 diabetes.

**Materials and Methods:**

A cross-sectional study was conducted. The study group comprised 15 patients with type 2 diabetes with asthma, and the control group comprised 145 patients with type 2 diabetes without asthma. Their fat distribution was evaluated by measuring the VFA by abdominal computed tomography at the umbilical level. Their glucose status was assessed by measuring the fasting plasma glucose (FPG) concentration, fasting immunoreactive insulin concentration, homeostasis model assessment (HOMA) index, and hemoglobin A1c concentration.

**Results:**

Among patients with type 2 diabetes, VFA was significantly greater in patients with asthma than those without asthma (*P* < 0.0001). The FPG concentration, fasting immunoreactive insulin concentration, and HOMA index were higher in patients with asthma than those without asthma (*P* < 0.05, *P* < 0.0001, and *P* < 0.0001, respectively). Multiple logistic regression analysis showed that VFA and the HOMA index were significantly associated with asthma in patients with type 2 diabetes (odds ratio, 1.78; 95% confidence interval, 1.31–3.89; *P* = 0.0115 and odds ratio, 3.65; 95% confidence interval, 1.37–7.85; *P* = 0.0078, respectively).

**Conclusions:**

Our data suggest that VFA and insulin resistance are associated with the development of asthma in patients with type 2 diabetes.

## 1. Introduction

The number of patients with type 2 diabetes has increased in Japan [1], and type 2 diabetes has attracted attention as a risk factor for asthma [2]. However, the risk factors for the development of asthma in patients with type 2 diabetes have not been determined.

Both asthma and obesity are common clinical problems that often coexist in the same patient, and epidemiologic studies have consistently demonstrated that obesity is a risk factor for asthma [3, 4]. Individuals with obesity have a higher prevalence of asthma, which tends to persist and require more intensive treatment in these patients [5–7]. In addition, many previous studies have shown that the strongest associations were present between asthma and measures of abdominal or visceral obesity [5, 7]. Furthermore, abdominal obesity is known to be a strong predictor of poor lung function [8].

Centrally located body fat is now generally considered to be more important than regional or generalized obesity in the development of metabolic syndrome. Metabolic syndrome comprises several risk factors for cardiovascular disease, including insulin resistance, hyperinsulinemia, dyslipidemia, obesity, diabetes mellitus, and hypertension [9, 10].

Although abdominal computed tomography (CT) can be used to precisely assess visceral fat accumulation (VFA), this technique is expensive; therefore, measurement of either the waist circumference or body mass index (BMI) is widely used [11]. VFA is a risk factor for cardiovascular disease [12] and is associated with insulin resistance in healthy individuals [13] and patients with type 2 diabetes mellitus [14]. Furthermore, several studies have suggested that insulin resistance may have a causative role in the development of asthma [15, 16], while patients with type 2 diabetes mellitus have a higher prevalence of asthma [2]. However, neither the risk factors for the development of asthma in patients with type 2 diabetes mellitus nor whether VFA and insulin resistance are associated with asthma in such patients has been determined.

We hypothesized that the presence of asthma is associated with VFA and insulin resistance in patients with type 2 diabetes. To test this hypothesis, we measured the blood pressure (BP), lipid concentrations, metabolic profile, and degree of VFA (using abdominal CT) in Japanese patients with type 2 diabetes either with or without asthma. We then determined the independent predictors of asthma in these patients.

## 2. Materials and Methods

### 2.1. Patients and Study Design

We screened 282 Japanese patients with type 2 diabetes mellitus who were consecutively admitted to Oita Red Cross Hospital from April 2007 to March 2017. Of these, 160 patients (83 men and 77 women) with a mean age of 56 ± 7 years fulfilled the inclusion criteria and were enrolled in the study. The inclusion criteria were as follows: no underlying causes of secondary hypertension (primary aldosteronism, renal vascular hypertension, hyperthyroidism, or pheochromocytoma); no history of chronic disease, such as renal failure (creatinine concentration of >1.5 mg/dl), pulmonary disease, liver dysfunction (aspartate aminotransferase concentration of >50 IU/l), arteriosclerosis obliterans, sleep apnea syndrome, or symptomatic cerebrovascular disease; not currently receiving treatment with insulin; and absence of organic heart disease according to treadmill exercise electrocardiography. Patients with an ST-T abnormality were also excluded.

In total, 122 of the screened patients were excluded from further analysis. Of those excluded, 52 patients were being treated with insulin, 18 had renal failure, 12 had angina pectoris, 10 had symptomatic cerebrovascular disease, 9 had sleep apnea syndrome, 8 had secondary hypertension (4 with primary aldosteronism, 2 with renal vascular hypertension, and 2 with hyperthyroidism), 7 had arteriosclerotic obliterans, and 6 had liver dysfunction (3 with hepatitis B and 3 with hepatitis C). The remaining 160 patients were enrolled in the study.

The study was conducted according to the principles in the Declaration of Helsinki. All patients provided written informed consent to participate in the study, and the study protocol was approved by the ethics committee of Oita Red Cross Hospital.

### 2.2. Definition of Allergic Disease and Use of Medication for Asthma

Asthma was considered to be present if the medical records contained a diagnosis of asthma made by a pulmonary specialist, based on a history of intermittent wheezing in combination with bronchodilator and/or methacholine responsiveness [17]. All 15 patients with asthma were using combination inhalers containing an inhaled corticosteroid and a long-acting *β*2 agonist. Nine of 15 patients were using salmeterol/fluticasone, and the remaining 6 were using budesonide/formoterol. Asthma attacks were well controlled using these inhaled corticosteroid/long-acting *β*2 agonist combinations. No patients had experienced severe asthma attacks within the previous few months, and none had been administered oral or systemic corticosteroids.

### 2.3. Definition of Hypertension

Essential hypertension was defined as a mean systolic BP of ≥140 mmHg, mean diastolic BP of ≥90 mmHg, or self-reported use of antihypertensive medication [18]. BP was measured three times by a mercury-column sphygmomanometer after 10 min of physical rest, and the mean value was calculated.

### 2.4. Laboratory Methods and Evaluation of Insulin Resistance

Blood was extracted from an antecubital vein with the patient in the recumbent position at 07:00, after an overnight fast. All patients underwent routine laboratory testing, including measurements of serum electrolytes, serum total cholesterol, serum triglycerides, serum high-density lipoprotein cholesterol (HDL-C), fasting plasma glucose (FPG), and fasting immunoreactive insulin (F-IRI). Dyslipidemia was defined as a fasting triglyceride level of ≥200 mg/dl or HDL-C concentration of <45 mg/dl for women and <35 mg/dl for men [18]. Insulin resistance was evaluated using the homeostasis model assessment (HOMA) index [19]: (fasting plasma insulin (*μ*U/ml) × FPG (mmol/l))/22.5.

### 2.5. VFA Measurement Using CT

All patients underwent CT for cross-sectional measurement of abdominal VFA at the umbilical level, determined using Fat scan version 3 software (N2 Systems, Osaka, Japan) as previously described [20]. This CT technique is widely used as a practical method for the evaluation of regional adiposity and has previously been validated [21].

### 2.6. Anthropometry and Body Composition

The patients' anthropometry and body composition were evaluated using the following parameters: height, body mass, BMI, and waist circumference. BMI was calculated as weight in kg/squared height in m. Waist circumference was measured midway between the lower rib and the iliac crest on the midaxillary line at the end of a gentle expiration with the patients in a standing position.

### 2.7. Statistical Analysis

All data are presented as mean ± standard deviation for two groups of patients: those with asthma and those without asthma (Table 1). For each variable in Table 1, the difference between the two groups was assessed using a two-sided test. Differences were considered statistically significant when *P* < 0.05. Student's *t-*test was used for continuous variables, and the chi-square test was used for categorical variables. Logistic regression analysis was used to assess the influence of explanatory variables on asthma, where the explanatory variables were age, sex, BMI, VFA, waist circumference, duration of diabetes, hypertension, dyslipidemia, BP, heart rate, total cholesterol, triglycerides, HDL-C, FPG, F-IRI, HOMA index, hemoglobin A1c, uric acid, C-reactive protein (CRP), and creatinine. Sex, hypertension, and dyslipidemia were dichotomized as 1 (presence) or 0 (absence) by cut-off values defined in the previous section. The presence of asthma was represented as 1, and the absence of asthma was represented as 0. A backward elimination procedure was employed to identify the significant factors among all the explanatory variables considered. All statistical analyses were performed using a standard statistical package (JMP 6.0; SAS Institute, Cary, NC, USA).

## 3. Results

Patients with type 2 diabetes mellitus were classified into two groups based on the presence or absence of asthma. Fifteen patients with type 2 diabetes (9.4%) had asthma and 145 (90.6%) did not.

As shown in Table 1, the degree of VFA was higher in patients with asthma than those without asthma (*P* < 0.0001). The BMI and waist circumference were also higher in patients with asthma than those without asthma (*P* = 0.0075 and *P* = 0.0005, respectively). The mean ages of the patients in each group were similar, and there were no significant differences in sex, the duration of diabetes, or the medication administered between the two groups.

With regard to lipid metabolism, the serum triglyceride concentration was higher and the serum HDL-C concentration was lower in patients with asthma than those without asthma (*P* = 0.0232 and *P* < 0.0001, respectively), whereas the serum total cholesterol concentration was similar in the two groups. With regard to glucose metabolism, the FPG concentration, fasting insulin concentration, and HOMA index were higher in patients with asthma than those without asthma (*P* = 0.0103, *P* < 0.0001, and *P* < 0.0001, respectively).

The serum uric acid concentration was higher in patients with asthma than those without asthma (*P* = 0.0060). However, there were no significant differences in the CRP or hemoglobin A1c concentration. Regarding renal function, there was no significant difference in the serum creatinine concentration between the groups.

Univariate logistic regression analysis showed that a higher risk of asthma was associated with a higher BMI (odds ratio (OR), 1.23; 95% confidence interval (CI), 1.04–1.53; *P* = 0.0126), VFA (OR, 2.16; 95% CI, 1.26–5.27; *P* < 0.0001), waist circumference (OR, 1.11; 95% CI, 1.03–1.18; *P* = 0.0025), and triglyceride concentration (OR, 1.02; 95% CI, 1.00–1.04; *P* = 0.0275); a lower HDL-C concentration (OR, 0.87; 95% CI, 0.82–0.97; *P* = 0.0049); and a higher FPG concentration (OR, 1.06; 95% CI, 1.01–1.10; *P* = 0.0138), F-IRI (OR, 4.46; 95% CI, 2.56–8.99; *P* < 0.0001), HOMA index (OR, 5.98; 95% CI, 2.18–14.80; *P* < 0.0001), and uric acid concentration (OR, 1.75; 95% CI, 1.14–2.62; *P* = 0.0075). All of these were dependent lipid and glucose metabolic parameters in patients with type 2 diabetes (Table 2).

Conversely, the multivariate logistic analysis identified VFA (OR, 1.78; 95% CI, 1.31–3.89; *P* = 0.0115) and the HOMA index (OR, 3.65; 95% CI, 1.37–7.85; *P* = 0.0078) as significant independent risk factors for asthma in patients with type 2 diabetes (Table 3).

## 4. Discussion

The risk factors for the development of asthma in patients with type 2 diabetes mellitus have not been determined. We hypothesized that the presence of asthma would be associated with VFA and insulin resistance in patients with type 2 diabetes. Measurement of metabolic parameters revealed that the serum HDL-C level was lower while the HOMA index and the degree of VFA were higher in patients with asthma than those without asthma. Multivariate logistic analysis revealed that substantial VFA and insulin resistance are independent risk factors for the presence of asthma in patients with type 2 diabetes. This is the first study to show that VFA evaluated by abdominal CT and insulin resistance are associated with asthma in such patients.

The association between obesity and asthma has been investigated in several large-scale prospective studies [22, 23]. These studies showed that the relative risk of incident asthma increased with an increasing BMI. In addition, a randomized controlled trial conducted in Finland revealed that low-calorie diets and exercise programs result in weight loss and concurrent control of asthma in overweight adults [24]. Furthermore, previous studies have shown that measures of abdominal or visceral obesity have the strongest associations with asthma [7]. Thus, in the present study, VFA was evaluated by abdominal CT to more accurately define the relationship between abdominal or visceral obesity and asthma.

Although the specific mechanism linking obesity and asthma remains to be elucidated, several mechanisms could explain our observations. First, obesity affects immune function [25]. Because the immune system is critically involved in the pathophysiology of asthma, some of these changes in immune function may be linked to asthma. Specifically, previous reports have hypothesized that the mechanical and adipokine-related effects of visceral adiposity cause or worsen asthma [6]. Second, obesity may be associated with a state of low-grade inflammation [26] that may contribute to airway inflammation [27]. A recent study showed that Th2-mediated inflammation and high-fat-diet–induced expression of chitinase 3-like 1 (a chitinase-like protein, also called YKL-40 in humans and breast regression protein-39 in rodents) simultaneously contribute to the genesis of both obesity and asthma [28].

Several reports have indicated that asthma is associated with insulin resistance in obese children, adolescents, and adults [15, 16]. Morbidly obese children and adolescents with asthma have a higher prevalence of insulin resistance than morbidly obese children and adolescents without asthma [15]. In addition, insulin resistance is associated with impaired lung function in adolescents, particularly in those who are overweight or obese [29]. In adults, insulin resistance also modifies the association between obesity and existing asthma; the association between these concurrent pathologies becomes stronger as the degree of insulin resistance increases [16].

The underlying pathophysiological mechanisms of the association between asthma and insulin resistance are not understood. In our opinion, several possible mechanisms exist. One is that both asthma and insulin resistance are derived from a generalized proinflammatory state. Data from animal and human studies have shown that obesity leads to a systemic proinflammatory state as indicated by higher plasma concentrations of various inflammatory markers, including CRP, interleukin 6 (IL-6), tumor necrosis factor *α* (TNF-*α*), and monocyte chemoattractant protein 1 (MCP-1) [30–33]. We previously reported that VFA is significantly associated with insulin resistance in patients with type 2 diabetes [34]. In addition, several reports have indicated that high circulating concentrations of inflammatory mediators (including CRP, IL-6, TNF-*α*, and MCP-1) originate from obese adipose tissue and that the levels of these mediators are associated with the degree of insulin resistance [35, 36]. Moreover, elevated concentrations of IL-6 and TNF-*α* in the bronchoalveolar lavage fluid of patients with asthma and elevated concentrations of plasma MCP-1 in patients with concurrent asthma and type 2 diabetes have been observed, and a relationship between these cytokines and asthma, including airway inflammation and remodeling, has been reported [37, 38].

The present study has several limitations. First, we studied a relatively small sample, and the study was cross-sectional in design. Sixty-three percent of the patients with asthma and 67% of the patients without asthma had been previously diagnosed with essential hypertension. All of these patients were being treated with one or more antihypertensive drugs, including angiotensin-converting enzyme inhibitors, angiotensin II receptor blockers, and calcium channel antagonists, prior to enrollment. All three of these drug classes have been reported to ameliorate insulin resistance [39, 40]; therefore, use of these medications might have skewed our findings. In addition, a considerable number of patients were being treated with sulfonylureas and/or alpha glucosidase inhibitors, and one patient in each group was being treated with pioglitazone, an insulin-sensitizing drug that has been shown to reduce VFA in patients with type 2 diabetes [41]. Nonetheless, the results indicate that the presence of asthma in patients with diabetes is associated with VFA and insulin resistance. The second limitation is that the patients' lung function was not assessed using spirometry. FEV_1_ is an important measure of asthma control [42]. Further studies are required to examine the relationships among FEV_1_, VFA, insulin resistance, and the presence of asthma in patients with type 2 diabetes. Finally, several reports have indicated that rhinitis, sinusitis, and gastroesophageal reflux disease are associated with the development or severity of asthma [43]. In the present study, we did not assess patients for rhinitis, sinusitis, or gastroesophageal reflux disease. A large-scale study is needed to clarify the relationships among these pathologies, various metabolic parameters, and the presence of asthma in patients with type 2 diabetes.

In conclusion, our findings suggest that asthma in patients with type 2 diabetes is associated with both abdominal VFA and insulin resistance. Large cohort studies, including studies of other populations, should be conducted in an attempt to corroborate these findings.

## Figures and Tables

**Table 1:** Patients' clinical characteristics.

	Without asthma	With asthma	*P* value
Age (years)	56 ± 8 (45-65)	55 ± 5 (43-65)	ns
Gender (men/women) (86/74)	75/70	8/7	ns
Visceral fat accumulation (cm^2^) (range = 37.2-208.6)	77 ± 21 (37.2-126.0)	155 ± 47 (58.5-208.6)	<0.0001
Duration of diabetes (years) (range = 0.83-20)	7.7 ± 4.6 (0.83-20.0)	8.1 ± 5.0 (1.1-16.7)	ns
Hypertension (%)	63	67	ns
Dyslipidemia (%)	38	40	ns
Smoking (%)	23	27	ns
Drug use (%)			
Sulfonylurea	37	33	ns
Alpha-glucosidase inhibitors	32	33	ns
Statin	30	33	ns
Calcium channel antagonists	39	40	ns
ACE inhibitors	23	20	ns
Angiotensin receptor blocker	32	33	ns
BMI (kg/m^2^) (range)	25.6 ± 2.9 (19.7-35.0)	27.4 ± 3.3 (22.6-33.1)	0.0071
Waist circumference (cm) (range)	84.5 ± 8.6 (68.3-102.3)	92.7 ± 10.7 (72.9-117)	0.0003
Systolic blood pressure (mmHg) (range)	130 ± 12 (110-152)	133 ± 10 (108-156)	ns
Diastolic blood pressure (mmHg) (range)	76 ± 8 (60-86)	77 ± 9 (62-88)	ns
Heart rate (bpm) (range)	68 ± 9 (54-78)	70 ± 8 (60-80)	ns
Total cholesterol (mg/dl) (range)	203 ± 34 (144-256)	210 ± 40 (125-267)	ns
Triglyceride (mg/dl) (range)	134 ± 37 (65-225)	151 ± 35 (95-189)	0.0212
HDL cholesterol (mg/dl) (range)	47 ± 9 (28-62)	41 ± 7 (30-47)	<0.0001
Fasting plasma glucose (mg/dl) (range)	138 ± 20 (102-183)	148 ± 27 (126-158)	0.0093
Fasting immunoreactive insulin (*μ*U/ml) (range)	6.5 ± 2.7 (2.4-9.3)	9.4 ± 2.2 (7-12.2)	<0.0001
HOMA index (range)	2.0 ± 0.6 (0.92-3.25)	3.4 ± 0.7 (2.3-4.37)	<0.0001
Hemoglobin A1c (%) (range)	7.6 ± 1.3 (6.1-10.3)	7.7 ± 0.8 (6.8-8.8)	ns
C-reactive protein (mg/dl) (range)	0.30 ± 0.16 (0.15-0.87)	0.38 ± 0.19 (0.17-0.76)	ns
Uric acid (mg/dl) (range)	6.1 ± 1.7 (3.2-9.0)	6.8 ± 1.2 (5.7-8.8)	0.0087
Creatinine (mg/dl) (range)	0.8 ± 0.2 (0.4-1.1)	0.9 ± 0.2 (0.6-1.0)	ns

Data are presented as mean ± standard deviation. ACE = angiotensin-converting enzyme; BMI = body mass index; HDL = high-density lipoprotein; HOMA = homeostasis model assessment; ns = not significant.

**Table 2:** Univariate logistic regression analysis of the relationship between asthma (dependent variable) and other parameters in patients with type 2 diabetes.

	Asthma		
	Odds ratio	95% CI	*P* value
Age	0.95	0.85-1.06	ns
Gender (men)	2.02	0.73-5.51	ns
BMI	1.23	1.04-1.53	0.0126
Visceral fat accumulation	2.16	1.26-5.27	<0.0001
Waist circumference	1.11	1.03-1.18	0.0025
Duration of diabetes	1.04	0.91-1.16	ns
Hypertension	1.24	0.47-3.48	ns
Dyslipidemia	1.18	0.42-3.16	ns
Smoking	1.10	0.61-2.92	ns
Systolic blood pressure	1.04	0.99-1.09	ns
Diastolic blood pressure	1.03	0.97-1.10	ns
Heart rate	1.02	0.95-1.12	ns
Total cholesterol	1.01	0.99-1.04	ns
Triglyceride	1.02	1.00-1.04	0.0275
High-density lipoprotein cholesterol	0.87	0.82-0.97	0.0049
Fasting plasma glucose	1.06	1.01-1.10	0.0138
Fasting immunoreactive insulin	4.46	2.56-8.99	<0.0001
Homeostasis model assessment	5.98	2.18-14.8	<0.0001
Hemoglobin A1c	1.15	0.82-1.92	ns
C-reactive protein	1.12	0.87-2.15	ns
Uric acid	1.75	1.14-2.62	0.0075
Creatinine	3.10	0.18-19.8	ns

Sex (female = 0, men = 1), hypertension (absent = 0, present = 1), and dyslipidemia (absent = 0, present = 1) were investigated as potential predictors of asthma. CI = confidence interval; BMI = body mass index.

**Table 3:** Multivariate logistic regression analysis, with asthma as the dependent variable, in patients with type 2 diabetes.

	Asthma		
	Odds ratio	95% CI	*P* value
VFA	1.78	1.31-3.89	0.0115
HOMA index	3.65	1.37-7.85	0.0078

CI = confidence interval; VFA = visceral fat accumulation; HOMA = homeostasis model assessment.

## Data Availability

The data that support the findings of this study are available from the corresponding author upon reasonable request.
